# Preclinical antitumor evaluation of a tetrahydrocannabinol and cannabidiol (1:6) cannabis extract in an MCF-7 xenograft model of estrogen receptor-positive breast cancer

**DOI:** 10.14202/vetworld.2026.2496-2511

**Published:** 2026-06-20

**Authors:** Nuntana Meesiripan, Somchai Thanasitthichai, Suleeporn Sangrajrang, Nuntakan Suwanpidokkul, Piyaporn Prayakprom, Chatchada Bodhibukkana, Vipada Khaowroongrueng, Kankanit Suriyachan, Attasit Srisubat, Pattamaporn Surawongsin, Kasem Rattanapinyopituk, Siriwan Sakarin

**Affiliations:** 1 Division of Research and Academic Support, National Cancer Institute, Bangkok, Thailand; 2 The Government Pharmaceutical Organization, Bangkok, Thailand; 3 Institute of Medical Research and Technology Assessment, Ministry of Public Health, Nonthaburi, Thailand; 4 Division of Medical Technical and Academic Affairs, Ministry of Public Health, Nonthaburi, Thailand; 5 Research and Technology Assessment Department, Ophthalmology Department, Lerdsin Hospital, Bangkok, Thailand; 6 Center of Excellence for Companion Animal Cancer, Department of Pathology, Faculty of Veterinary Science, Chulalongkorn University, Bangkok, Thailand; 7 Biochemistry Unit, Department of Physiology, Faculty of Veterinary Science, Chulalongkorn University, Bangkok, Thailand

**Keywords:** apoptosis, breast cancer, cannabidiol, cannabinoids, estrogen receptor-positive, MCF-7, tetrahydrocannabinol, xenograft model

## Abstract

**Background and Aim:**

Breast cancer remains one of the leading causes of cancer-related mortality worldwide, despite advances in surgery, chemotherapy, endocrine therapy, and targeted treatments. Cannabinoids derived from *Cannabis sativa*, particularly tetrahydrocannabinol (THC) and cannabidiol (CBD), have demonstrated anticancer properties in several experimental models; however, *in vivo* evidence in estrogen receptor (ER)-positive breast cancer remains limited. This study aimed to evaluate the antitumor effects of a THC:CBD (1:6) cannabis extract in a Michigan Cancer Foundation-7 breast cancer cell line (MCF-7) xenograft mouse model of ER-positive breast cancer.

**Materials and Methods:**

Female BALB/c nude mice bearing MCF-7 xenograft tumors were randomly assigned into five groups (n = 5/group): negative control (sesame oil), positive control treated with 5-fluorouracil (5-FU; 20 mg/kg), and three treatment groups receiving oral THC:CBD (1:6) extract at doses of 2, 10, or 20 mg/kg body weight for 30 consecutive days. Tumor growth was monitored throughout the experiment. Histopathological examination and immunohistochemical analysis of proliferating cell nuclear antigen (PCNA) expression were performed to evaluate apoptosis-related morphology and tumor cell proliferation. Hematological and biochemical parameters were assessed to determine systemic safety.

**Results:**

Cannabinoid-treated groups exhibited significant suppression of tumor growth compared with the negative control group. Tumor volume reduction was observed in all treatment groups, with the greatest reduction detected in the high-dose THC:CBD group. Histopathological evaluation revealed increased numbers of tumor cells exhibiting morphological features consistent with apoptosis in cannabinoid-treated mice. Immunohistochemical analysis demonstrated significantly lower PCNA expression scores in all THC:CBD-treated groups compared with both negative and positive controls, indicating reduced tumor cell proliferation. Hematological parameters remained within normal physiological ranges in cannabinoid-treated animals. However, elevated alanine aminotransferase and aspartate aminotransferase levels were observed in the high-dose group, suggesting potential dose-related hepatic stress.

**Conclusion:**

The THC:CBD (1:6) cannabis extract demonstrated significant antitumor activity in an MCF-7 xenograft model by suppressing tumor progression primarily through inhibition of tumor cell proliferation, with supportive apoptosis-related histological features. These findings provide novel *in vivo* evidence supporting the potential of cannabinoid-based formulations as adjunctive therapeutic approaches for ER-positive breast cancer.

## INTRODUCTION

Breast cancer is one of the most common cancers in women worldwide and remains a leading cause of cancer-related mortality. According to hospital-based cancer registries, it continues to be the most prevalent cancer among women in Thailand, with incidence rates rising in parallel with global trends [1, 2]. Despite advances in screening and treatment, mortality rates remain high. The prognosis strongly depends on the stage at diagnosis, with early-stage cancers generally associated with more favorable outcomes than advanced-stage disease [3]. Management of breast cancer typically requires a multidisciplinary approach, incorporating surgery, radiotherapy, systemic chemotherapy, endocrine therapy, and targeted biological therapies, often used in combination depending on tumor subtype and stage. Treatment decisions are largely guided by the expression of estrogen receptor (ER), progesterone receptor (PR), and human epidermal growth factor receptor 2 (HER2). However, receptor status can change over time, which may significantly affect therapy selection. Triple-negative breast cancers (TNBC), lacking all three receptors, generally have a poorer prognosis and rely primarily on chemotherapy, with few targeted therapeutic options [4, 5].

While these treatments have improved survival, they are frequently associated with adverse effects such as alopecia, nausea, vomiting, and immunosuppression, which can negatively impact quality of life [3, 6]. Consequently, there is a pressing need to identify novel, safer, and more effective therapeutic strategies. In recent years, herbal-based medicinal compounds, such as cannabis, have attracted attention as potential alternative or adjunct therapies for cancer patients. Cannabis contains several bioactive compounds, the main ones being cannabinoids, including delta-9-tetrahydrocannabinol (THC) and cannabidiol (CBD), which are responsible for most of its biological effects [7]. Cannabinoids have been reported not only to reduce symptoms associated with cancer or its treatments, such as nausea, vomiting, pain, and loss of appetite, but also to enhance the efficacy of conventional chemotherapeutic agents while mitigating their side effects [8, 9]. Beyond these palliative effects, preclinical studies indicate that cannabinoids can directly inhibit tumor progression by targeting multiple stages, including proliferation, angiogenesis, invasion, and metastasis, as well as inducing apoptosis and autophagy [10, 11]. As mentioned above, breast cancer treatment is largely guided by receptor status, which can change over time and potentially limit the effectiveness of receptor-dependent therapies. In contrast, cannabinoids appear to exert antitumor effects independently of receptor expression. This is supported by recent *in vitro* meta-analytic evidence showing that CBD potently inhibits breast cancer cell growth regardless of receptor status, suggesting a receptor-independent mechanism of action [12]. These findings are further supported by *in vivo* studies in mouse xenograft models using MDA-MB-231 TNBC cells, in which both CBD and THC individually reduced primary tumor growth, suppressed lung metastasis, and prolonged survival [13].

Although the anticancer properties of cannabinoids have been increasingly reported in both *in vitro* and *in vivo* studies, several important gaps remain in the current literature. Most previous breast cancer studies have focused on either THC or CBD as single agents, while limited evidence is available regarding the combined use of these cannabinoids in defined ratios [14]. In addition, most *in vivo* breast cancer studies have been conducted using TNBC models, particularly MDA-MB-231 xenografts, whereas studies investigating ER-positive breast cancer models remain scarce. This is clinically important because ER-positive breast cancer represents one of the most common subtypes in women and exhibits distinct biological behavior and therapeutic responses compared with TNBC. Furthermore, the antitumor effects of whole-cannabis extracts containing multiple phytocannabinoids have not been adequately evaluated in Michigan Cancer Foundation-7 breast cancer cell line (MCF-7) breast cancer xenograft models. Existing studies have also provided limited comparisons between cannabinoid-based treatments and conventional chemotherapeutic agents such as 5-fluorouracil (5-FU), restricting the translational relevance of the findings [15]. Moreover, the influence of a pharmacokinetically optimized THC:CBD ratio on tumor proliferation and apoptosis-related responses in receptor-positive breast cancer remains poorly understood. Therefore, further *in vivo* investigation is required to clarify the therapeutic potential, biological effects, and safety profile of combined cannabinoid formulations in ER-positive breast cancer models.

This study aimed to evaluate the antitumor effects of a defined-ratio THC:CBD (1:6) whole-cannabis extract in an MCF-7 xenograft mouse model of ER-positive breast cancer. Specifically, the study investigated the effects of the cannabinoid extract on tumor growth, histopathological alterations, apoptosis-related morphology, and tumor cell proliferation by analyzing proliferating cell nuclear antigen (PCNA) expression. In addition, the study assessed hematological and biochemical parameters to determine the treatment’s systemic safety profile. The therapeutic efficacy of the cannabinoid extract was also compared with the conventional chemotherapeutic agent 5-FU to provide clinically relevant preclinical evidence regarding the potential use of cannabinoid-based formulations as adjunctive therapeutic approaches for ER-positive breast cancer.

## MATERIALS AND METHODS

### Ethical approval

All experimental procedures involving animals were reviewed and approved by the Institutional Animal Care and Use Committee of the National Cancer Institute, Thailand (Protocol No. 272_2019RB_IN602), and Lerdsin Hospital, Department of Medical Services (Protocol No. AEC-F-v03-02). All animal experiments were conducted in accordance with relevant institutional guidelines and regulations for the care and use of laboratory animals. The study also complied with the ARRIVE 2.0 guidelines for reporting animal research. Humane endpoints were established before study initiation and included severe weight loss, impaired mobility, ulceration or infection at the tumor site, or signs of severe clinical deterioration [16]. Animals were monitored regularly throughout the experimental period to minimize pain and distress. No animals reached the predefined humane endpoints during the study.

### Study period and location

The study was conducted at the accredited animal research facility of Lerdsin Hospital, Department of Medical Services, Bangkok, Thailand. Cell culture experiments and xenograft preparation were performed in collaboration with the Faculty of Veterinary Science, Chulalongkorn University, Bangkok, Thailand. Cannabinoid extracts were prepared and supplied by the Government Pharmaceutical Organization (GPO), Thailand.

### Study design

Female BALB/cAJcl-nu nude mice, four weeks of age, were obtained from Nomura Siam International (Bangkok, Thailand). Animals were housed under controlled environmental conditions (22 ± 2°C, 50%–70% humidity, 12 h light/dark cycle) with free access to food and water. A one-week acclimatization period was provided before experimentation.

To support estrogen-dependent tumor growth, mice received weekly subcutaneous injections of 17β-estradiol prepared from β-estradiol powder (Sigma E2758, Sigma-Aldrich, St. Louis, MO, USA). Estradiol was initially dissolved in sterile sesame oil to a stock concentration of 2 mg/mL and subsequently diluted to deliver 5 µg per 0.1 mL per mouse. The solution was freshly prepared weekly and administered subcutaneously under aseptic conditions. Injections began 1 week before tumor cell inoculation and continued weekly throughout the study period [17]. However, serum estradiol concentrations were not measured.

MCF-7 breast cancer cells at a concentration of 1 × 10^7^ viable cells were mixed with Matrigel (Corning, NY, USA) at a 1:1 ratio (v/v) and subcutaneously injected into the right flank of each mouse under aseptic conditions. Body weight (BW) and tumor dimensions were recorded every 3 days. Tumor length and width were measured using a caliper, and tumor volume was calculated using the following formula: Tumor volume (mm³) = 1/2 (length × width²) [18].

Mice were randomly assigned to treatment groups once tumors became established, defined as the presence of a palpable tumor mass at the injection site approximately 19 days post-inoculation, using simple randomization. Tumor sizes were comparable among groups at baseline (day 0). Blinding was not performed because treatment groups received different doses.

The sample size was determined using *a priori* power analysis performed in Minitab (Minitab LLC, State College, PA, USA). Based on an effect size (f = 0.827) derived from a previous study [19], with a significance level of 0.05, statistical power of 0.80, and five experimental groups, the minimum total sample size required to detect significant differences was calculated to be 25 animals. Accordingly, 25 mice (n = 5 per group) were included in the study. This sample size is consistent with previous preclinical studies using cannabinoid-based treatments, in which 5–8 animals per group were sufficient to detect significant differences in tumor growth, proliferation, and apoptosis [20].

The animals were randomly allocated into five groups: (1) negative control group receiving sesame oil by oral gavage, (2) positive control group treated intraperitoneally with 5-FU (20 mg/kg) three times weekly, and (3–5) experimental groups receiving cannabinoid extract (THC:CBD, 1:6) supplied by the GPO at doses of 2, 10, or 20 mg/kg BW by oral gavage for 30 consecutive days. All animals with successfully established tumors were included in the study. Three animals died during the experimental period, including one mouse each from groups 1–3. One animal displayed severe weight loss, one was found dead without an apparent cause, and one developed rectal prolapse. These events were considered incidental and unrelated to tumor burden or treatment. No additional animals or data points were excluded from the final analysis.

### Cannabinoid formulation

The GPO, Thailand, prepared and supplied a THC and CBD solution at a 1:6 ratio using *Cannabis sativa* L. cultivated in the GPO medical cannabis greenhouse. The extraction process followed modified methods from previous studies [21]. Briefly, cannabis flowers (cola) were harvested, and cannabinoids were isolated using cold ethanol extraction. After quantification of THC and CBD concentrations, the extract was adjusted to achieve the desired THC:CBD ratio of 1:6.

The selection of this ratio was based on pharmacokinetic considerations to achieve approximately balanced systemic exposure. In rodent pharmacokinetic studies, oral administration of THC and CBD at equivalent doses showed that THC remained detectable in plasma at later time points, whereas CBD concentrations declined more rapidly and became undetectable at the same time point, indicating faster systemic clearance of CBD than THC *in vivo* [22]. In addition, previous studies in mice demonstrated that CBD inhibits cytochrome P450-mediated metabolism of THC, resulting in a prolonged half-life and increased persistence of THC and its metabolites, particularly in brain tissue, compared with THC administered alone [23]. These findings indicate that co-administration of THC and CBD alters the pharmacokinetic profiles of both compounds. Consistent with these observations, co-administration studies in experimental animals have reported reduced maximum serum concentrations of CBD and increased THC exposure compared with single-compound administration.

Oral administration of the whole-cannabis extract was selected to better reflect potential clinical application compared with intraperitoneal administration of single compounds. Therefore, a THC:CBD ratio of 1:6 was selected to compensate for the faster clearance of CBD and the altered metabolism of THC, with the aim of achieving an approximately 1:1 effective systemic exposure of THC and CBD *in vivo*, comparable to the ratio used in the *in vitro* cell culture experiments (unpublished data). Following extraction, the solvent was removed by rotary evaporation, and the concentrated cannabinoid extract was dissolved in pharmaceutical-grade oil for *in vivo* administration. The stock solution was prepared at a concentration of 5 mg/mL and further diluted in sesame oil to achieve the desired dosing concentrations based on BW (mg/kg). All experiments were conducted using the same production batch of the extract to minimize variability.

Exact concentrations of individual cannabinoids beyond THC and CBD, as well as full compositional profiling, including terpene content, residual solvents, and stability data, were not available. Therefore, the presence of additional constituents in the extract cannot be excluded. However, the extract was produced under standardized conditions by the GPO in Thailand to ensure batch consistency and quality control.

### *In vitro* screening for animal dose selection

A cell viability assay was performed to assess the antiproliferative effects of cannabis extracts, building on previous studies conducted in multiple cancer cell lines. A cannabis extract with a 1:1 THC:CBD ratio was used for the *in vitro* experiments. In contrast to *in vivo* conditions, *in vitro* experiments do not involve pharmacokinetic processes such as absorption, distribution, metabolism, and elimination; therefore, a 1:1 ratio was directly applied to reflect the intended effective exposure at the cellular level.

The half-maximal inhibitory concentration (IC50), defined as the concentration required to reduce cell proliferation by 50%, was determined to guide dose selection for subsequent *in vivo* studies. In MCF-7 breast cancer cells, the IC50 of this extract was approximately 1 µg/mL (unpublished data). Dose selection for the xenograft study was based on a combination of *in vitro* potency (IC50) and *in vivo* pharmacokinetic considerations, with dose calculations performed in consultation with pharmacists from the GPO. Based on *in vivo* pharmacokinetic data (unpublished), oral administration of THC at 10 mg/kg was estimated to achieve a maximum blood concentration of approximately 0.4 µg/mL in rodents. To achieve a target systemic concentration closer to the *in vitro* IC50 value (~1 µg/mL), a higher dose was therefore required. Accordingly, 20 mg/kg was selected as the high-dose level, and 2 and 10 mg/kg were selected as the low- and intermediate-dose levels, respectively.

### Cell line expansion and preparation for transplantation

The human breast cancer cell line MCF-7 (CRL-3435™, American Type Culture Collection [ATCC], Manassas, VA, USA) was cultured in Dulbecco’s Modified Eagle Medium (DMEM) (ATCC™ 30-2002, ATCC), supplemented with 10% fetal bovine serum (cell-culture tested, ATCC™ 30-2020, ATCC), in accordance with the guidelines provided by ATCC. Cells were maintained in 25 cm³ sterile culture flasks (NUNC EasyFlask, Thermo Scientific, Shanghai, China) under standard conditions at 37°C in a humidified incubator with 5% CO_2_. Cells were used at low passage numbers (<20 passages) to maintain biological consistency. Short tandem repeat profiling and mycoplasma testing were not performed; however, culture conditions were maintained in accordance with ATCC guidelines to minimize variability.

The medium was renewed every 3 days to maintain nutrient balance and cell health. Upon reaching 70%–80% confluence, adherent cells were detached using 0.25% Trypsin-EDTA solution (Gibco, Thermo Scientific, Waltham, MA, USA), pelleted by centrifugation, and resuspended in fresh medium. Expanded cultures were subsequently transferred into 75 cm³ flasks for large-scale propagation until adequate cell numbers were obtained for transplantation.

For xenograft preparation, cells were harvested, washed twice with sterile phosphate-buffered saline (PBS, pH 7.2, Gibco), and resuspended in complete DMEM. Cell concentration and viability were assessed using the 0.4% Trypan blue staining method and counted using a hemocytometer. The suspension was then adjusted to 1 × 10^7^ viable cells in 0.1 mL for subcutaneous injection into the right flank of nude mice.

### Histopathological evaluation

At the end of the 30-day treatment period, mice were euthanized by carbon dioxide inhalation in a dedicated chamber in accordance with institutional animal care guidelines. Animals were exposed to carbon dioxide until respiration ceased and death was confirmed. Blood samples were collected for hematological and biochemical evaluation. Tumors and major internal organs were excised, fixed in 10% neutral-buffered formalin for 24 h, and embedded in paraffin. Tissue sections were stained with hematoxylin and eosin (H&E) to evaluate histopathological features, detect potential metastases, and quantify necrotic and apoptotic areas following procedures described previously [24].

Briefly, the percentage of necrotic area was calculated by dividing the necrotic region by the total tumor area using an image analysis system (NIS-Elements Analysis D, Nikon, Tokyo, Japan). Apoptotic cells were manually counted in five randomly selected high-power fields (HPFs) at 40× magnification, and the results were reported as the average number of apoptotic cells per field.

### Immunohistochemistry

Tumor cell proliferation was assessed using immunohistochemistry. Proliferation was evaluated using a monoclonal mouse anti-PCNA antibody (clone PC10, Dako, Hamburg, Germany) at a dilution of 1:400. For each mouse, five HPF at 40× magnification were randomly selected for analysis. PCNA immunoreactivity was scored according to staining intensity and the proportion of positive cells as follows: 0 = no staining, 1 = weak staining or 0%–25% positive cells, 2 = 26%–50% positive cells, 3 = 51%–75% positive cells, and 4 = >75% positive cells [25].

### Statistical analysis

Statistical analysis was conducted using SPSS software version 22.0 (IBM, Chicago, IL, USA). Before statistical comparisons, data were assessed for normality using the Shapiro-Wilk test, and normally distributed values were presented as mean ± standard deviation. Differences among groups were analyzed using one-way analysis of variance followed by Tukey’s post hoc test for multiple comparisons. A p < 0.05 was considered statistically significant.

## RESULTS

### BW and tumor growth

The mean BW of mice across all five groups before the experiment showed no significant differences (p = 0.832). By day 30, at the conclusion of the study, BWs remained comparable among groups (p = 0.848). All groups exhibited an overall increase in BW during the experimental period, with no significant differences observed among groups (p = 0.693) (Table 1).

**Table 1:** The average body weight (BW) of mice in each group at the start of the experiment (D0) and on day 30 (D30), together with the percentage change in BW.

Group	D0 (g)	D30 (g)	BW change (%)
Negative control	22.79 ± 0.99	24.69 ± 0.61	8.67 ± 2.38
Positive control (5-FU)	22.74 ± 0.50	25.56 ± 0.51	12.49 ± 2.90
Low-dose THC:CBD (2 mg/kg BW)	22.35 ± 0.30	25.09 ± 0.58	12.23 ± 2.28
Intermediate-dose THC:CBD (10 mg/kg BW)	22.88 ± 0.45	25.05 ± 0.65	9.43 ± 1.31
High-dose THC:CBD (20 mg/kg BW)	23.31 ± 0.49	25.61 ± 0.76	9.86 ± 2.30

Data are expressed as mean ± SD. 5-FU = 5-fluorouracil, THC: CBD = tetrahydrocannabinol:cannabidiol, SD = Standard deviation.

**Figure 1:**
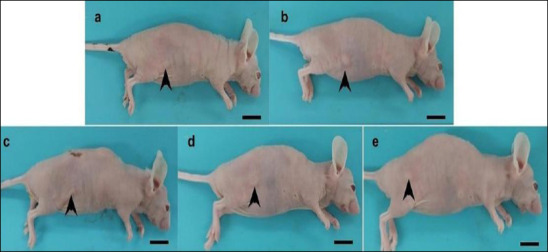
Gross appearance of tumors in mice subcutaneously injected with 1 × 10^7^ MCF-7 cells. (a) Negative control, gavaged with sesame oil; (b) positive control, treated with 5-fluorouracil (5-FU, 20 mg/kg, intraperitoneally, three times per week); (c) THC:CBD (1:6) low-dose group, gavaged at 2 mg/kg BW; (d) THC:CBD (1:6) intermediate-dose group, gavaged at 10 mg/kg BW; (e) THC:CBD (1:6) high-dose group, gavaged at 20 mg/kg BW. Scale bar = 1 cm. Tumor masses were located subcutaneously and appeared as small nodules at the sites indicated by the arrows. 5-FU = 5-fluorouracil, BW = Body weight, THC: CBD = tetrahydrocannabinol:cannabidiol, SD = Standard deviation.

**Figure 2:**
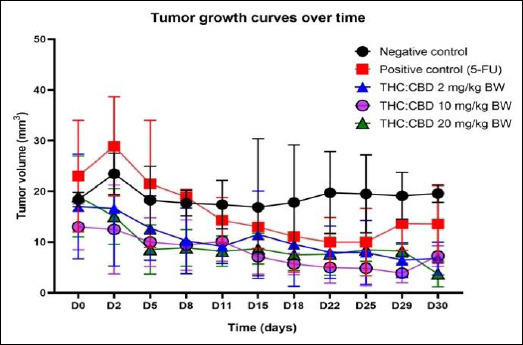
Tumor growth curves over time in different treatment groups. Tumor volume (mm³) was measured every 3 days following treatment initiation. Data are presented as mean ± standard deviation.

The mean tumor volume of mice in each group before treatment also showed no significant differences (p = 0.477) (Table 2). By day 30, tumors in all experimental groups had substantially decreased in size, making them difficult to detect visually, although they remained palpable on physical examination (Figure 1). At this point, the mean tumor volume differed significantly among the groups (p < 0.001) (Table 2). Specifically, the mean tumor volumes in the positive control group (5-FU) and in the cannabinoid-treated groups (THC:CBD, 1:6) at low (2 mg/kg BW), intermediate (10 mg/kg BW), and high (20 mg/kg BW) doses were significantly smaller than those in the negative control group (p = 0.043, p < 0.001, p < 0.001, and p < 0.001, respectively). Furthermore, compared with the positive control group, tumor volumes in the cannabinoid-treated groups were also significantly reduced (p = 0.023, p = 0.026, and p = 0.001 for the low-, intermediate-, and high-dose groups, respectively). However, no significant differences were observed among the cannabinoid-treated groups (p = 0.120) (Table 2). Tumor growth curves showed a progressive reduction in tumor volume in the treatment groups compared with the negative control group (Figure 2). All treatment groups showed sustained suppression of tumor growth throughout the study period.

**Table 2:** The average tumor volume of mice in each group at the start of the experiment (D0) and on day 30 (D30), together with the percentage change in tumor volume.

Group	D0 (mm³)	D30 (mm³)	Tumor volume change (%)
Negative control	18.38 ± 0.71	19.56 ± 0.87	7.34 ± 8.40
Positive control (5-FU)	23.00 ± 5.50	13.58 ± 3.76^a^	-35.99 ± 12.90^a^
Low-dose THC:CBD (2 mg/kg BW)	17.09 ± 5.16	6.75 ± 1.61^a,b^	-51.88 ± 14.65^a^
Intermediate-dose THC:CBD (10 mg/kg BW)	13.18 ± 2.02	7.26 ± 0.91^a,b^	-39.71 ± 12.39^a^
High-dose THC:CBD (20 mg/kg BW)	19.23 ± 4.38	3.75 ± 1.16^a,b^	-75.94 ± 11.31^a,b^

Data are expressed as mean ± SD. 5-FU = 5-fluorouracil, THC: CBD = tetrahydrocannabinol:cannabidiol, SD = Standard deviation.

a Indicates significantly different from the negative control group (p < 0.05).

b Indicates significantly different from the positive control group (5-FU) (p < 0.05).

**Figure 3:**
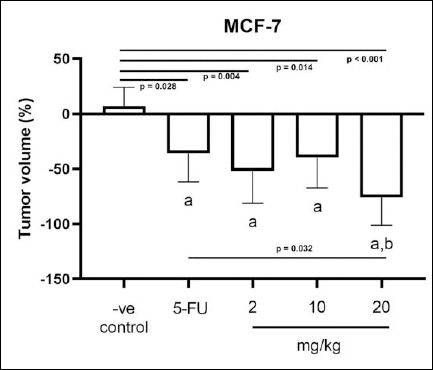
Percentage change in tumor volume of mice in the negative control group, positive control group (5-FU), and cannabinoid-treated groups (THC:CBD, 1:6) at doses of 2, 10, and 20 mg/kg BW. Data are presented as mean ± SD. ^a^ Indicates significantly different from the negative control group (p < 0.05). ^b^ Indicates significantly different from the positive control group (5-FU) (p < 0.05). 5-FU = 5-fluorouracil, THC: CBD = tetrahydrocannabinol:cannabidiol, SD = Standard deviation.

Analysis of the percentage change in tumor volume showed a slight increase in the negative control group, whereas the positive control group and all cannabinoid-treated groups exhibited reductions. These reductions were statistically significant compared with the negative control group (p = 0.028, p = 0.004, p = 0.014, and p < 0.001, respectively). Comparisons between the cannabinoid-treated groups and the positive control group revealed further tumor volume reductions in the cannabinoid groups; however, statistical significance was achieved only in the high-dose group (p = 0.032), while no significant differences were observed in the low-dose (p = 0.391) and intermediate-dose groups (p = 0.831). The detailed results are presented in Table 2 and Figure 3.

### Hematological and clinical chemistry analysis

No significant differences were observed in hematological and clinical chemistry parameters between the cannabinoid-treated groups and the negative control group. However, mice in the positive control group (5-FU) exhibited significantly lower red blood cell (RBC) counts and hemoglobin levels, and higher mean corpuscular volume (MCV) compared with the other groups (p < 0.001), although all values remained within the normal physiological range. Other clinical chemistry values did not differ significantly among groups; however, mice receiving the high-dose THC:CBD extract (20 mg/kg BW) showed significantly elevated alanine aminotransferase (ALT) and aspartate aminotransferase (AST) levels compared with the other groups (p < 0.001), exceeding typical physiological ranges and suggesting a potential effect on liver function (Table 3).

**Table 3:** Hematological and clinical chemistry profiles of mice

Parameter	Unit	Negative control	Positive control (5-FU)	Low-dose THC:CBD (2 mg/kg BW)	Intermediate-dose THC:CBD (10 mg/kg BW)	High-dose THC:CBD (20 mg/kg BW)
RBC count	×10⁶ cell	9.7 ± 0.15	9.0 ± 0.24^a^	9.7 ± 0.08^b^	9.9 ± 0.17^b^	9.7 ± 0.15^b^
Hct	%	45.0 ± 1.15	43.3 ± 0.96	45.0 ± 0.82	45.4 ± 0.89	45.3 ± 1.53
Hb	g/dL	15.3 ± 0.57	14.6 ± 0.22	15.3 ± 0.14	15.8 ± 0.19^b^	15.8 ± 0.21^b^
MCV	fL	46.3 ± 0.50	47.9 ± 0.30^a^	46.1 ± 0.70^b^	45.8 ± 0.50^b^	46.5 ± 0.20
MCH	pg	15.6 ± 0.26	16.2 ± 0.14	15.7 ± 0.26	15.7 ± 0.30	15.9 ± 0.23
MCHC	g/dL	34.0 ± 0.35	33.8 ± 0.21	34.0 ± 0.19	34.4 ± 0.29	34.1 ± 0.46
RDW	%	17.7 ± 0.42	18.1 ± 0.39	17.5 ± 0.32	18.1 ± 0.69	18.0 ± 0.25
Platelet	×10⁶ cell	1.34 ± 0.115	1.42 ± 0.112	1.23 ± 0.334	1.16 ± 0.395	1.32 ± 0.103
WBC count	cell	3,175 ± 814	3,175 ± 1,271	4,450 ± 2,016	4,400 ± 354	4,267 ± 1,012
Neutrophil	%	49.5 ± 5.20	40.8 ± 13.43	48.0 ± 10.92	36.8 ± 6.42	45.7 ± 1.53
Lymphocyte	%	49.5 ± 5.20	56.5 ± 11.33	50.5 ± 10.38	61.0 ± 6.82	51.0 ± 1.73
Monocyte	%	1.0 ± 0.00	2.3 ± 2.50	1.3 ± 0.50	1.4 ± 0.55	1.7 ± 0.58
Eosinophil	%	0.0 ± 0.00	0.5 ± 1.00	0.25 ± 1.00	0.8 ± 1.00	1.7 ± 2.00
Basophil	%	0.0 ± 0.00	0.0 ± 0.00	0.0 ± 0.00	0.0 ± 0.00	0.0 ± 0.00
ALT	U/L	43.8 ± 13.84	49.8 ± 5.12	47.3 ± 5.12	74.0 ± 62.71	305.8 ± 389.89^a^,^b^
AST	U/L	210.3 ± 10.41	194.0 ± 8.08	222.0 ± 34.60	272.8 ± 112.49	643.4 ± 616.05^a^,^b^
BUN	mg/dL	29.0 ± 5.66	28.0 ± 4.08	29.3 ± 6.18	26.4 ± 2.61	29.6 ± 2.30
Creatinine	mg/dL	0.6 ± 0.00	0.6 ± 0.10	0.6 ± 0.00	0.6 ± 0.04	0.6 ± 0.04
Albumin	g/dL	3.2 ± 0.30	2.9 ± 0.15	3.5 ± 0.30	3.5 ± 0.15	3.9 ± 0.81
Total protein	g/dL	7.5 ± 0.52	7.0 ± 0.26	7.8 ± 0.24	8.0 ± 0.34	8.7 ± 1.19

Data are expressed as mean ± SD.

a Indicates significantly different from the negative control group (p < 0.05).

b Indicates significantly different from the positive control group (5-FU) (p < 0.05).

RBC = Red blood cell, Hct = Hematocrit, Hb = Hemoglobin, MCV = Mean corpuscular volume, MCH = Mean corpuscular hemoglobin, MCHC = Mean corpuscular hemoglobin concentration, RDW = Red blood cell distribution, ALT = Alanine aminotransferase, AST = Aspartate aminotransferase, BUN = Blood urea nitrogen. 5-FU = 5-fluorouracil, THC: CBD = tetrahydrocannabinol:cannabidiol, SD = Standard deviation.

### Macroscopic and histopathological morphology

**Figure 4:**
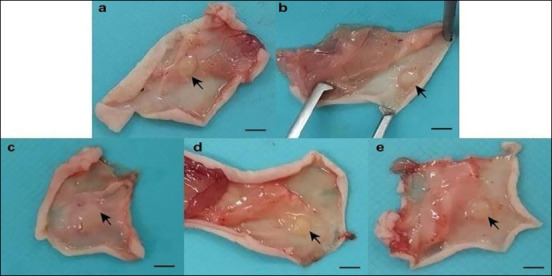
Gross appearance of tumors in mice subcutaneously injected with 1 × 10^7^ MCF-7 cells. (a) Negative control, gavaged with sesame oil; (b) positive control, treated with 5-fluorouracil (5-FU, 20 mg/kg, intraperitoneally, three times per week); (c) THC:CBD (1:6) low-dose group, gavaged at 2 mg/kg BW; (d) THC:CBD (1:6) intermediate-dose group, gavaged at 10 mg/kg BW; (e) THC:CBD (1:6) high-dose group, gavaged at 20 mg/kg BW. Scale bar = 0.5 cm. Tumors appeared as small, firm, well-circumscribed nodules at the injection sites (arrows). 5-FU = 5-fluorouracil, THC: CBD = tetrahydrocannabinol:cannabidiol.

Subcutaneous tumor masses developed at the injection sites in all five experimental groups, although the tumors were relatively small. The masses were generally well-circumscribed, firm, and nodular, allowing detection by palpation despite their limited size (Figures 1 and 4). Tumor growth remained localized to the injection sites, and no gross abnormalities or evidence of metastasis to other internal organs, including the thoracic and abdominal cavities, were observed in any mice.

**Figure 5:**
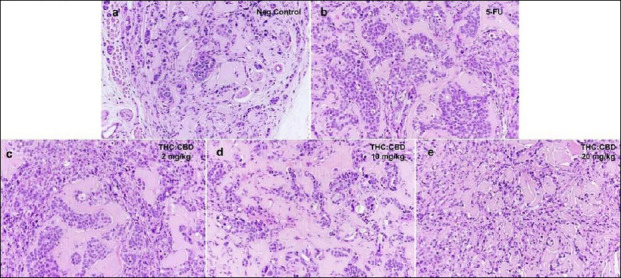
Microscopic histopathology of subcutaneous tumors in mice injected with Michigan Cancer Foundation-7 breast cancer cell line and subsequently administered (a) negative control, gavaged with sesame oil; (b) positive control, treated with 5-fluorouracil (5-FU, 20 mg/kg, intraperitoneally, three times per week); (c) THC:CBD (1:6) low-dose group, gavaged at 2 mg/kg BW; (d) THC:CBD (1:6) intermediate-dose group, gavaged at 10 mg/kg BW; (e) THC:CBD (1:6) high-dose group, gavaged at 20 mg/kg BW. Sections were stained with hematoxylin and eosin (H&E) and examined at 40× magnification. 5-FU = 5-fluorouracil, BW = Body weight, THC: CBD = tetrahydrocannabinol:cannabidiol.

**Figure 6:**
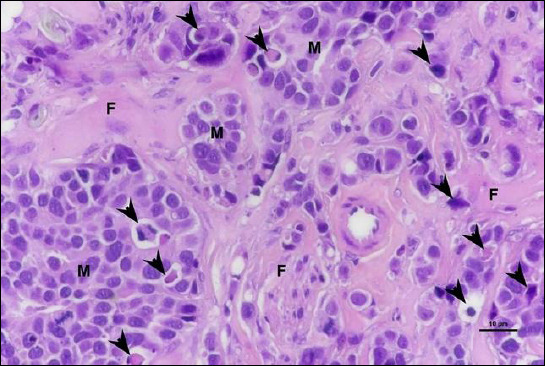
Histopathology of adenocarcinoma tumor nodules (M) from mice subcutaneously injected with Michigan Cancer Foundation-7 breast cancer cell line and treated with cannabinoid extract revealed apoptotic tumor cells (arrows). The tumors predominantly exhibited a lobular growth pattern, with individual lobules separated by fibrous connective tissue (F). Sections were stained with hematoxylin and eosin (H&E) and examined at 40× magnification.

**Figure 7:**
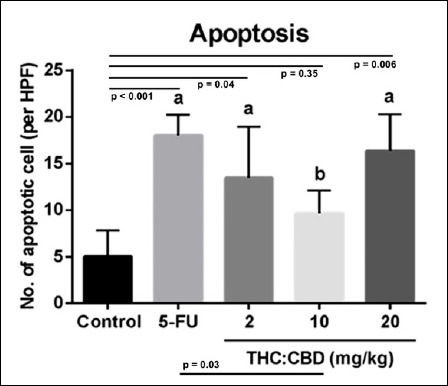
Apoptotic cell counts per high-power field in tumor tissues of mice from the negative control group, positive control group (5-FU), and cannabinoid-treated groups (THC:CBD, 1:6) at doses of 2, 10, and 20 mg/kg BW. Data were expressed as mean ± SD. ^a^ Indicates significantly different from the negative control group (p < 0.05). ^b^ Indicates significantly different from the positive control group (5-FU) (p < 0.05). 5-FU = 5-fluorouracil, THC: CBD = tetrahydrocannabinol:cannabidiol, SD = Standard deviation.

At the microscopic level, all groups exhibited subcutaneous tumor nodules composed of adenocarcinoma cells (Figure 5). The tumors predominantly displayed a lobular architecture, with individual lobules separated by fibrous connective tissue resembling stromal elements (Figure 6). No distinct necrotic regions were observed in any group, and histopathological examination did not reveal evidence of tumor cell dissemination to other organs. Higher magnification revealed apoptotic cells distributed throughout the tumor mass (Figure 6). The mean apoptotic cell counts were significantly higher in the positive control group (5-FU) (p < 0.001) and in the cannabinoid-treated groups receiving the low-dose (2 mg/kg BW) (p = 0.040) and high-dose (20 mg/kg BW) (p = 0.006) compared with the negative control group. Interestingly, the intermediate-dose group (10 mg/kg BW) exhibited significantly fewer apoptotic cells per HPF than the positive control group (p = 0.030) (Table 4, Figure 7).

**Table 4:** The average apoptotic cell counts per high-power field in tumor tissues.

Group	Average apoptotic cells/HPF
Negative control	5.00 ± 1.414
Positive control (5-FU)	18.00 ± 1.115^a^
Low-dose THC:CBD (2 mg/kg BW)	13.47 ± 3.171^a^
Intermediate-dose THC:CBD (10 mg/kg BW)	9.62 ± 1.238^b^
High-dose THC:CBD (20 mg/kg BW)	16.33 ± 2.293^a^

Data were expressed as mean ± SD. 5-FU = 5-fluorouracil, THC: CBD = tetrahydrocannabinol:cannabidiol, SD = Standard deviation.

a Indicates significantly different from the negative control group (p < 0.05).

b Indicates significantly different from the positive control group (5-FU) (p < 0.05).

### PCNA expression

**Figure 8:**
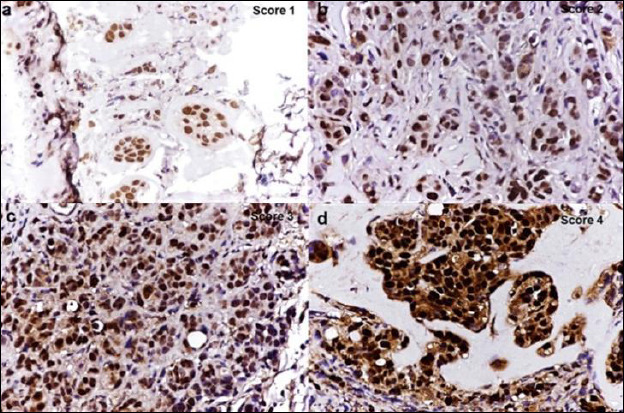
Proliferating cell nuclear antigen expression in tumor cells from mice subcutaneously injected with Michigan Cancer Foundation-7 breast cancer cell line: (a) score = 1, (b) score = 2, (c) score = 3, and (d) score = 4 (counterstained with Mayer’s hematoxylin, 40×).

**Figure 9:**
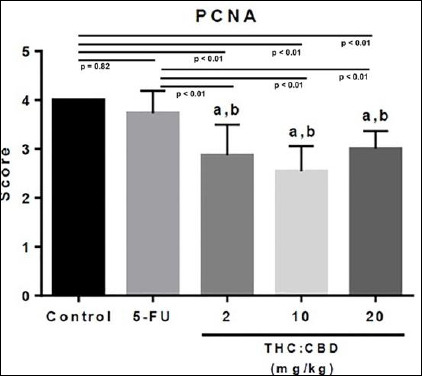
Average histological scores of proliferating cell nuclear antigen (PCNA)-positive tumor cells relative to the total number of tumor cells per high-power field (HPF) in the negative control group, positive control group (5-FU), and cannabinoid-treated groups (THC:CBD, 1:6) at doses of 2, 10, and 20 mg/kg BW. Data were expressed as mean ± SD. ^a^ Indicates significantly different from the negative control group (p < 0.05). ^b^ Indicates significantly different from the positive control group (5-FU) (p < 0.05). 5-FU = 5-fluorouracil, BW = Body weight, THC: CBD = tetrahydrocannabinol: cannabidiol, SD = Standard deviation.

PCNA-positive cells were observed in tumors from all groups, with expression scores ranging from 1 to 4 (Figure 8). No significant difference in PCNA expression was detected between the negative control and positive control (5-FU) groups (p = 0.820). In contrast, all cannabinoid-treated groups, including low-, intermediate-, and high-doses of THC:CBD, showed significantly lower PCNA expression compared with both the negative control (p < 0.001, p < 0.001, and p < 0.001, respectively) and positive control groups (p < 0.001, p < 0.001, and p < 0.001, respectively) (Table 5, Figure 9).

**Table 5:** Expression scores of proliferating cell nuclear antigen (PCNA)-positive tumor cells

Group	PCNA score
Negative control	4.00 ± 0.000
Positive control (5-FU)	3.73 ± 0.467
Low-dose THC:CBD (2 mg/kg BW)	2.87 ± 0.626^a,b^
Intermediate-dose THC:CBD (10 mg/kg BW)	2.54 ± 0.519^a,b^
High-dose THC:CBD (20 mg/kg BW)	3.00 ± 0.366^a,b^

Data were expressed as mean ± SD. 5-FU = 5-fluorouracil, THC: CBD = tetrahydrocannabinol:cannabidiol, SD = Standard deviation.

a Indicates significantly different from the negative control group (p < 0.05).

b Indicates significantly different from the positive control group (5-FU) (p < 0.05).

## DISCUSSION

Cannabinoids present in cannabis have been reported to modulate multiple cellular processes involved in cancer progression, including apoptosis induction and inhibition of cell proliferation, migration, and angiogenesis [10, 11]. The principal bioactive compounds, CBD and THC, exert anticancer effects through distinct molecular mechanisms [26], suggesting that their combined administration may enhance antitumor efficacy compared with single-compound treatment [11, 14]. Previous studies have shown that cannabis extracts suppress proliferation and promote apoptotic cell death in various cancer cell types [13, 27]; however, *in vivo* investigations focusing on combined THC and CBD treatment in ER-positive breast cancer models remain limited. The present study demonstrated that a THC:CBD (1:6) extract suppressed tumor growth in the MCF-7 breast cancer xenograft model, primarily by inhibiting tumor cell proliferation, with supportive apoptosis-related histological features and no clear evidence of systemic toxicity, except for elevated liver enzymes in the high-dose group.

### BW and systemic safety

BW was monitored as a general indicator of systemic health during treatment. Previous studies examining the effects of cannabinoids on BW have reported inconsistent results: some describe no significant changes following CBD administration [28–30], whereas others report weight reduction associated with decreased food intake or increased activity following CBD treatment [31–34]. In the present study, no significant differences in BW were observed among mice treated with different doses of the THC:CBD extract compared with controls, suggesting that the treatment did not markedly affect overall health or nutritional status during the experimental period.

Hematological and clinical chemistry parameters were further evaluated to assess systemic safety. As expected, mice treated with 5-FU exhibited hematological alterations consistent with early myelosuppressive effects [35]. In contrast, no significant hematological changes were detected in any THC:CBD-treated group. However, mice receiving the high-dose THC:CBD extract (20 mg/kg BW) showed significantly elevated ALT and AST levels beyond the normal physiological range, indicating a potential effect on liver function at this dose. This finding is consistent with previous reports demonstrating hepatotoxic effects of high-dose CBD in animal models, potentially mediated through impaired glutathione homeostasis [36, 37]. However, no overt histopathological abnormalities were observed in the examined tissues, suggesting that these changes may reflect early or mild biochemical alterations rather than definitive liver injury. In addition, a comprehensive toxicity assessment, including organ weight measurement, was not fully performed in this study and represents a limitation. These results highlight the importance of defining safe dosing ranges and further investigating the mechanisms underlying cannabinoid-associated hepatotoxicity.

### Tumor growth and xenograft model considerations

The antitumor efficacy of the THC:CBD (1:6) extract was evaluated using a subcutaneous MCF-7 xenograft model in BALB/c nude mice, a widely used platform for preclinical assessment of anticancer therapies due to reduced immune-mediated tumor rejection [38–42]. To facilitate tumor establishment, a relatively high number of MCF-7 cells were transplanted in combination with Matrigel [43, 44]. Although palpable tumors developed within 2–4 weeks, tumor volumes remained relatively small throughout the study. This observation is consistent with previous reports indicating that ER-positive breast cancer cell lines such as MCF-7 often require extended periods to achieve substantial tumor growth *in vivo* [39].

Given that the MCF-7 model represents an ER-positive breast cancer subtype, tumor growth is largely dependent on estrogen signaling pathways. Emerging evidence suggests that cannabinoids may interact with hormone-related signaling, including modulation of ER activity, which could influence tumor cell proliferation in ER-positive cancers [45, 46]. Therefore, the antiproliferative effects observed in this study may, in part, be associated with interactions within ER-dependent regulatory mechanisms. However, these findings may be specific to ER-positive tumor biology, and whether similar effects occur in ER-negative breast cancer models remains to be determined. Further mechanistic studies are required to clarify the relationship between cannabinoid activity and hormone signaling pathways in different breast cancer subtypes. These findings should be considered preliminary, as the current study primarily demonstrates an antiproliferative effect and does not establish mechanistic causality. Further studies incorporating detailed molecular and pathway-level analyses are required to better define the mechanisms underlying the observed effects of combined THC:CBD treatment. It should be noted that serum estradiol levels were not measured, and single-agent THC or CBD effects were not evaluated, which may limit the mechanistic interpretation of the observed antiproliferative effects.

Small tumor size can complicate accurate volume measurement with caliper-based methods [47] and may limit the sensitivity to detect treatment-related differences. Despite consistent measurement by a single investigator, variability related to tumor shape and subcutaneous tissue may have contributed to this limitation. Future studies may benefit from optimizing xenograft conditions by increasing cell numbers, adjusting the Matrigel-to-cell ratio, extending the interval between transplantation and treatment initiation, or employing more profoundly immunodeficient mouse strains. In addition, estrogen supplementation may further enhance tumor establishment, as MCF-7 cells are ER-positive and estrogen support has been shown to improve xenograft growth [48, 49]. In the present study, estrogen supplementation was provided through weekly subcutaneous injections of 17β-estradiol to support tumor establishment. Although this approach has been used in rodent hormone studies [19] and was sufficient to support tumor formation in this model, continuous-release methods such as estrogen pellets or silastic implants are generally preferred for maintaining stable, sustained systemic hormone levels in MCF-7 xenograft models [48]. In this study, serum estradiol levels were not measured, and intermittent injections may have resulted in fluctuating or suboptimal estrogen exposure. This could have contributed to the relatively small tumor size observed and may have influenced tumor growth kinetics and sensitivity for detecting treatment effects. Therefore, incorporation of continuous-release estrogen supplementation or use of alternative models, such as orthotopic implantation, may improve physiological relevance and tumor growth consistency, thereby enabling more accurate evaluation of treatment effects. Nevertheless, histopathological examination confirmed the presence of cancer cells in all experimental groups, validating successful tumor establishment in this model.

Tumor weight was not determined at the study endpoint, which may have provided a more reliable measure of tumor burden, particularly in the context of small tumor volumes. Given the small absolute tumor size, measurement variability may have influenced the observed differences; therefore, the results should be interpreted with caution when distinguishing biological effects from potential measurement artifacts. Although a trend toward greater tumor suppression was observed at higher doses, no statistically significant dose-dependent differences were detected. This may reflect limited statistical power to detect subtle differences among dose groups, particularly given the relatively small sample size and small tumor burden in this model. Therefore, these findings should be interpreted cautiously. Future studies with larger sample sizes and optimized tumor models may help to better resolve potential dose-response relationships.

### Apoptosis-related morphology and PCNA expression

Histopathological analysis revealed an increased presence of tumor cells exhibiting morphological features consistent with apoptosis, including nuclear condensation and fragmentation, in the treatment groups compared with the negative control group. These findings are consistent with previous reports describing apoptosis-related changes following cannabinoid treatment in breast cancer cells [50, 51]. However, because apoptosis was evaluated primarily by histological criteria, these findings should be interpreted as indicative of an apoptotic trend rather than definitive mechanistic evidence. It should also be noted that MCF-7 cells lack functional caspase-3 expression, which limits the use of cleaved caspase-3 as a marker for detecting apoptosis in this model [52]. Incorporation of complementary assays, such as Terminal deoxynucleotidyl transferase dUTP nick end labeling (TUNEL) staining, Annexin V labeling, or evaluation of apoptosis-related molecular markers, such as the Bax/Bcl-2 ratio, would provide a more comprehensive assessment of apoptotic activity in future studies [53, 54].

Cannabinoids have been widely reported to exert antiproliferative effects in various cancer models, including breast cancer, through suppression of cell cycle progression and tumor cell growth [26, 55]. In the present study, tumor cell proliferation was assessed using PCNA [56]. Although PCNA-positive cells were detected across all groups, THC:CBD treatment significantly reduced PCNA expression compared with both control groups, indicating a clear antiproliferative effect consistent with previous reports [57, 58]. This reduction in proliferation likely represents a primary mechanism underlying the observed suppression of tumor growth.

### Limitations and future perspectives

Several limitations of this study should be acknowledged. The lack of detailed analytical characterization of the THC:CBD extract, including full compositional profiling and assessment of potential additional constituents due to its proprietary nature, may limit reproducibility and comparability with other studies. In addition, the study did not include single-agent THC or CBD treatment arms, which limits the ability to distinguish the individual contributions of each compound to the observed antitumor effects. Plasma concentrations of THC and CBD were not measured, and pharmacokinetic data would be useful for correlating systemic exposure with observed biological effects. Furthermore, the absence of blinding and the relatively small sample size may introduce potential bias and limit statistical power, particularly for subjective assessments such as histopathological evaluation and PCNA scoring. Short tandem repeat authentication and mycoplasma testing of the MCF-7 cell line were not performed, which may affect the reliability and reproducibility of the findings, although the cells were obtained from a commercial supplier and maintained under standard culture conditions.

Further studies incorporating optimized strategies for estrogen supplementation, comprehensive analytical profiling, pharmacokinetic validation, and more rigorous experimental design will be important for strengthening the robustness and translational relevance of these findings. Finally, caution should be exercised when extrapolating these preclinical results to clinical breast cancer, as xenograft models may not fully recapitulate human tumor biology, treatment response, or pharmacokinetics.

## CONCLUSION

In conclusion, the present study demonstrated that the THC:CBD (1:6) whole-cannabis extract exerted significant antitumor activity in an MCF-7 xenograft model of ER-positive breast cancer. Treatment with the cannabinoid extract resulted in marked suppression of tumor growth, significant reduction in tumor volume, decreased PCNA expression, and increased numbers of tumor cells exhibiting apoptosis-related morphological features. Among the tested doses, the high-dose THC:CBD group showed the greatest reduction in tumor volume, indicating a strong antiproliferative effect of the cannabinoid formulation. Importantly, no major hematological abnormalities were observed in cannabinoid-treated animals, although elevated ALT and AST levels in the high-dose group suggested potential dose-related hepatic stress.

These findings support the potential application of cannabinoid-based formulations as adjunctive therapeutic approaches for ER-positive breast cancer. The use of a pharmacokinetically optimized THC:CBD ratio and comparison with the conventional chemotherapeutic agent 5-FU provides clinically relevant preclinical evidence regarding the therapeutic potential of combined cannabinoids. In addition, the study contributes novel *in vivo* evidence regarding the effects of a whole-cannabis extract in receptor-positive breast cancer, an area that remains insufficiently explored.

A major strength of this study was the use of a defined-ratio whole-cannabis extract, evaluated in an ER-positive xenograft model, alongside histopathological, immunohistochemical, hematological, and biochemical assessments.

Overall, despite the inherent limitations of xenograft models, the present findings indicate that the THC:CBD (1:6) cannabis extract possesses promising antiproliferative and antitumor properties in ER-positive breast cancer. These results provide a foundation for future mechanistic and translational studies exploring cannabinoids as potential adjunctive agents in breast cancer therapy.

## DATA AVAILABILITY

The datasets generated and analyzed during the current study are available from the corresponding author upon reasonable request.

## AUTHORS’ CONTRIBUTIONS

NM: Cell culture, animal model experiments, data analysis and interpretation, and manuscript drafting and revision. ST, SSan, and AS: Conceptualization and supervision. NS, PP, CB, and VK: Preparation of cannabinoid solutions and supervision of related experiments. KS: Cell culture, laboratory work, and data analysis. PS: Supervision of research and laboratory animal studies. KR: Animal experiments, data analysis and interpretation, and manuscript editing and revision. SSak: Animal experiments, data analysis and interpretation, manuscript drafting, and approval of the submitted version. All authors have read and approved the final manuscript.
